# Stepwise analysis of *MIR9* loci identifies miR-9-5p to be involved in Oestrogen regulated pathways in breast cancer patients

**DOI:** 10.1038/srep45283

**Published:** 2017-03-27

**Authors:** Raffaela Barbano, Barbara Pasculli, Michelina Rendina, Andrea Fontana, Caterina Fusilli, Massimiliano Copetti, Stefano Castellana, Vanna Maria Valori, Maria Morritti, Paolo Graziano, Ciuffreda Luigi, Michelina Coco, Francesco Picardo, Tommaso Mazza, Ella Evron, Roberto Murgo, Evaristo Maiello, Manel Esteller, Vito Michele Fazio, Paola Parrella

**Affiliations:** 1 Laboratory of Oncology IRCCS “Casa Sollievo della Sofferenza”, San Giovanni Rotondo (FG), Italy; 2 Unit of Biostatistics IRCCS “Casa Sollievo della Sofferenza”, San Giovanni Rotondo (FG), Italy; 3 Unit of Bioinformatics IRCCS “Casa Sollievo della Sofferenza”, San Giovanni Rotondo (FG), Italy; 4 Oncology Department IRCCS “Casa Sollievo della Sofferenza”, San Giovanni Rotondo (FG), Italy; 5 Pathology Unit IRCCS “Casa Sollievo della Sofferenza”, San Giovanni Rotondo (FG), Italy; 6 Breast Unit, IRCCS Casa Sollievo della Sofferenza, San Giovanni Rotondo, Italy; 7 Laboratory of Genetic and Clinical Pathology, University Campus Bio-Medico of Rome, Italy; 8 Assaf Harofeh Medical Center Zerifin, Affiliated with Tel Aviv University, Sakler School of Medicine, Israel; 9 Cancer Epigenetics and Biology Program (PEBC), Bellvitge Biomedical Research Institute (IDIBELL), L’Hospitalet de Llobregat, Barcelona, Catalonia, Spain; 10 Institució Catalana de Recerca i Estudis Avançats (ICREA), Barcelona, Catalonia, Spain; 11 Department of Physiological Sciences II, School of Medicine, University of Barcelona, Barcelona, Catalonia, Spain

## Abstract

miR-9 was initially identified as an epigenetically regulated miRNA in tumours, but inconsistent findings have been reported so far. We analysed the expression of miR-9-5p, miR-9-3p, pri-miRs and *MIR9* promoters methylation status in 131 breast cancer cases and 12 normal breast tissues (NBTs). The expression of both mature miRs was increased in tumours as compared to NBTs (P < 0.001) and negatively correlated with ER protein expression (*P* = 0.005 and *P* = 0.003, for miR-9-3p and miR-9-5p respectively). In addition, miR-9-5p showed a significant negative correlation with PgR (P = 0.002). Consistently, miR-9-5p and miR-9 3p were differentially expressed in the breast cancer subgroups identified by ER and PgR expression and HER2 amplification. No significant correlation between promoter methylation and pri-miRNAs expressions was found either in tumours or in NBTs. In the Luminal breast cancer subtype the expression of miR-9-5p was associated with a worse prognosis in both univariable and multivariable analyses. Ingenuity Pathway Analysis exploring the putative interactions among miR-9-5p/miR-9-3p, ER and PgR upstream and downstream regulators suggested a regulatory loop by which miR-9-5p but not miR-9-3p is induced by steroid hormone receptor and acts within hormone-receptor regulated pathways.

miR-9 (hsa-miR-9-5p, miR-9-5p) is a highly conserved microRNA (miRNA) primarily expressed in the central nervous system^1^,^2^. It displays a tissue-specific expression pattern in brain, and regulates its normal development in physiological conditions^1^,^2^,^3^,^4^,^5^. Although several evidences link aberrant miR-9 expression to cancer development and progression, its overall role in cancerogenesis as well as the putative association with cancer patients’ clinical outcome remain largely unclear.

In humans, miR-9 is transcribed from three independent genomic loci mapping to chromosomes 1q22 (*MIR9-1*), 5q14.3 (*MIR9-2*) and 15q26.1 (*MIR9-3*)^6^ whose primary transcripts ultimately give rise to two functional mature miRNAs, i.e. miR-9-5p and miR-9-3p^6^. Although less intensively investigated, also miR-9-3p has been implicated in the development of the central nervous system and recent reports have demonstrated that miR-9-5p/9-3p can cooperate in regulating the balance between self-renewal and differentiation of human neural stem cells^6^.

The promoters of *MIR9* loci are embedded within CpG islands, and hypermethylation in at least one of these regions has been reported in several tumour types such as breast cancer^7^,^8^,^9^,^10^,^11^,^12^,^13^, thus the best studied miR-9-5p has been designated as an epigenetically regulated miRNA. However, only a partial or none correlation between gene promoters methylation and miR-9-5p expression has been demonstrated in breast cancer^8^ and gastric tumours^14^, respectively so far. Furthermore, the expression levels of miR-9-5p resulted highly variable across different carcinomas, being down-regulated in gastric carcinoma^11^, ovarian cancer^15^,^16^, and malignant mesothelioma^17^, or overexpressed in brain tumours^18^, osteosarcoma^19^, and hepatocellular carcinoma^20^. Unfortunately, inconsistent, even opposite findings have been often reported in the same cancer type, including breast cancer^21^,^22^,^23^,^24^.

Conflicting evidences also exist about the functional role that miR-9-5p may play within the tumour context. For instance, MYC mediated overexpression of miR-9 in non-metastatic breast cancer cells enabled them to form pulmonary micrometastases in mice^25^, and the use of ‘miRNA sponges’ in malignant cells with high metastatic potentials was able to reduce metastases formation^25^. Moreover, two recent studies remarked that miR-9 expression mediates invasion and metastatic potential in triple negative breast cancer cell lines, and that exosome-mediated delivery of miR-9 induces cancer associated fibroblast-like properties in human breast fibroblasts^26^. By contrast, Selcuklu *et al*.^22^ found that increased miR-9 expression levels in breast cancer cells were able to induce anti-proliferative, anti-invasive and pro-apoptotic activity.

Less is known about the putative role of miR-9-3p in cancer. Decreased levels of miR-9-3p were significantly associated with a worse prognosis in colorectal cancer^27^. *In vitro*, miR-9-3p was identified as a tumour-suppressor miR targetting TAZ (PDZ-binding motif)/YAP (yes-associated protein) expression in HCC cells^28^ and β1 Integrin in claudin-low breast cancer cells thereby sensitizing MDA-MB-231 to MEK inhibition^29^.

However, although expressed at a similar level^6^, only few studies have reported so far about miR-9 at both 5p and 3p level, especially in a cancer set. In light of this, we took the effort of clarifying the role of miR-9-5p/3p in primary breast cancer tissues and of evaluating their potentials as clinical relevant prognostic biomarkers. To this aim, the expression of miR-9-5p, miR-9-3p, pri-miR-9-1, pri-miR-9-2 and pri-miR-9-2, and methylation status of the three gene promoter regions were evaluated in a prospectively collected retrospective cohort including 131 breast cancer cases (discovery cohort). As controls, 12 breast samples from reductive mammoplasty were analysed. We found that both miR-9-5p and miR-9-3p were overall up-regulated in tumours as compared with normal breast tissues. Moreover, mature miRNAs expression negatively correlated with Oestrogen Receptor (ER) expression in cancer cells, but only miR-9-5p expression showed a strong negative correlation with expression of Progesteron Receptor (PgR). The analysis of the TCGA breast cancer dataset indicated that miR-9 is differentially expressed in the molecular subclasses identified by PAM50 signatures^30^. Although promoter methylation in at least one of the *MIR9* promoters was detected in 90% of the tumours, it did not correlate with the expression of any of the pri-miRNAs. Finally, survival analyses demonstrated an association between miR-9-5p expression and survival in the surrogate luminal breast cancer subgroup.

## Results

### Patients and treatment

 Table 1 summarizes descriptive statistics for the 131 cases from the discovery cohort analyzed for miR-9-5p/3p expression. The median age at diagnosis of the study population is 59.8 years (interquartile range, IQR: 46.5–71.2). Metastases at diagnosis were present in 9 cases (7%) whereas, among non metastatic patients (n = 122), 41 (33.6%) experienced disease progression (5 only local recurrence, 25 only distant metastases, and 11 both), with a disease progression rate of 6.6 events per 100 person/years, and 34 patients died from the disease (27.9%), with a mortality rate of 4.9 events per 100 person/years.

All patients received adequate local treatment (breast conserving surgery or total mastectomy), plus sentinel node biopsy or complete axillary dissection, followed by radiation as indicated. Post-surgery systemic treatments were given according to the accepted guidelines: St. Gallen, NCCN and ASCO.

### miR-9-5p and miR-9-3p are overexpressed in tumour samples as compared with normal tissues from reductive mammoplasty

**Figure 1:**
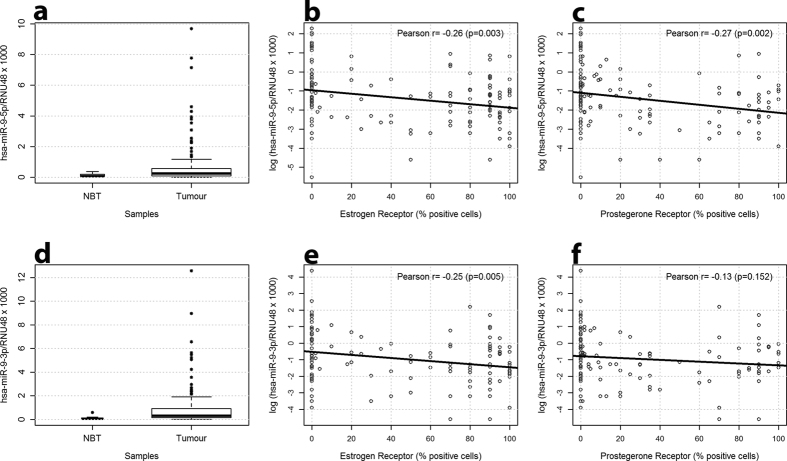
The evaluation of miR-9-5p expression in the discovery set by qPCR demonstrates the up-regulation of miR-9-5p in tumours and the negative correlation with hormone receptor status. (**a**) miR-9-5p is overall overexpressed in tumours (n = 131) as compared with normal breast tissues (n = 12) from reductive mammoplasty (p = 0.029); (**b**) miR-9-5p expression negatively correlates with the percentage of cells positive for Oestrogen Receptor (r = −0.26, p = 0.003) and (**c**) Progesterone Receptor (r = −0.27, p = 0.002); (**d**) miR-9-5p is differentially expressed in breast cancer subgroups characterized by ER positive or negative status (p = 0.001), (**e**) PgR positive or negative status (p = 0.002), and (**f**) molecular subtypes identified by ER positive status (Luminal), *HER2* amplification (HER2-amp), absence of both ER and PgR and negative for *HER2* amplification (Triple Negative) (p = 0.008).

The expression of miR-9-5p (hsa-miR-9-5p/RNU48 × 1000) and miR-9-3p (hsa-miR-9-3p/RNU48 × 1000) was evaluated in 131 and 122 breast cancers respectively and the normal breast tissues obtained from reductive mammoplasty (NBTs) (n = 12). In both cases expression levels were significantly higher in tumour samples as compared with normal breasts. For miR-9-5p the median expression was 0.25 (IQR: 0.09–0.58) in tumour samples and 0.07 (IQR: 0.06–0.19) in normal breast tissues (p = 0.029) (Fig. 1a). Similarly, the median expression for miR-9-3p was 0.34 (IQR: 0.15–0.95) in tumors and 0.06 (IQR: 0.04–0.13) in normal breast tissues (p = 0.004) (Fig. 1d). As expected, the expression of miR-9-3p positively correlated with the expression of miR-9-5p (Pearson correlation coefficient r = 0.41; p < 0.001) further suggesting that they are coordinately expressed in breast tissues.

### miR-9-5p up-regulation is associated with reduced ER and PgR expression in breast cancer

Then, we investigated the association between miR-9-5p and miR-9-3p expression and tumour clinicopathological characteristics. We found a negative correlation between miR-9-5p expression and the percentage of cells positive for ER (r = −0.26; P = 0.003) and PgR (r = −0.27; P = 0.002) (Fig. 1b,c). Whereas, miR-9-3p expression positively correlated with ki67/miB1 stained cells (r = 0.20, p = 0.042) and negatively correlated with age (r = −0.21 p = 0.022), tumour dimension (r = −0.21 p = 0.019) and ER expression (r = −0.25; p = 0.005). No significant correlation was found between miR-9-3p and percentage of PgR expressing cells (Fig. 1e,f).

When we compared cancer cases classified on the basis of surrogate molecular classification (Luminal, HER2 amplified and Triple Negative), the luminal subtype showed the lowest miR-9-5p levels (Median: 0.17, IQR: 0.07–0.40). By contrast, Triple Negative and HER2 positive tumours showed higher miR-9-5p expression levels (Median: 0.28 IQR: 0.16–3.54 and Median: 0.44, IQR: 0.22–0.55 respectively) (p = 0.008) (Table 1). No differences were found between Triple negative and HER2 amplified samples (data not shown). As regards miR-9-3p expression, similar levels were found in the luminal subtype and HER2 amplified subtype (Median: 0.28, IQR: 0.14–0.67 and Median: 0.24, IQR: 0.13–0.96 respectively), whereas the highest levels were detected in Triple Negative tumours (Median: 1.26, IQR: 0.36–2.59) (p = 0.004).

### miR-9-5p expression but not miR-9-3p expression is associated with survival in the luminal breast cancer subtype

The association with time-to-event outcomes (i.e. OS, PFS and MFS) was evaluated in the group of patients without metastases at diagnosis (n = 122). No statistically significant associations were found for either miR-9-5p or miR-9-3p in the overall population. However, in the luminal breast cancer subgroup (n = 76), miR-9-5p showed a significant association with OS, PFS and MFS in the intermediate expression group (i.e. II tertile: expression between 0.10 and 0.32) as compared with the low expression group (i.e. I tertile: expression lower than 0.10), both in univariable and multivariable Cox models (Table S2).

### The analysis of miR-9 expression in the TCGA breast cancer dataset confirms the association with hormone receptor status

**Figure 2:**
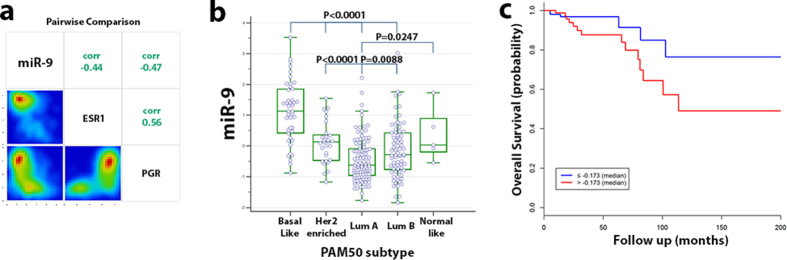
The analysis of the TCGA breast cancer data set (n = 256) demonstrates the differential expression of miR-9 in the molecular subgroups identified by the PAM50 signatures and the association between lower miR-9 expression and worse overall survival (n = 256). (**a**) miR-9 expression negatively correlates with Oestrogen and Progesterone receptors mRNA levels. miR-9 is differentially expressed in breast cancer subgroups characterize by (**b**) the PAM50 molecular subtypes. (**c**) Kaplan Meyer survival curves in cases without synchronous metastases (n = 243) indicate that patients with miR-9 expression levels above the median have a worse prognosis as compared with patients showing levels below the median.

We retrieved mRNA and miRNA expression data from the TCGA breast cancer dataset released in 2012 (PMID: 23000897)^31^. This dataset consists of a total of 825 samples, but global expression data of miRNAs and mRNAs, as well as information about ER and PgR receptors (ESR1 and PGR respectively), were available only for 256 cases (Table S3). Moreover, miRNA expression data do not specify which mature miRNA, i.e. miR-9-5p or miR-9-3p, they specifically refer to. Results from this analysis demonstrated a negative correlation between miR-9 expression and ER (r = −0.53;p < 0.001) and PgR (r = −0.45;p < 0.001) at RNA expression level (Fig. 2a).

Then, we evaluated the correlation between miR-9 and the molecular classification based on the PAM50 signature^30^ (n = 256). As shown in Fig. 2b, miR-9 was differentially expressed in the breast cancer molecular subgroups (p < 0.001 Kruskall-Wallis test), with the highest expression levels in the basal like and HER2 enriched PAM50 subtypes, and the lowest in the two luminal subtypes. In addition, as compared with our result on the discovery cohort, the analysis of the TCGA dataset identified statistically significant differences between basal like and HER2 enriched subtypes (p < 0.001 Mann-Whitney test). Moreover, miR-9 was differentially expressed between Luminal A and Luminal B subtypes (p = 0.009 Mann-Whitney test).

Due to the lack of data regarding PFS and MFS in the TCGA dataset, only the association between miR-9 and overall survival could be evaluated in 243 cases without synchronous metastases. In univariable Cox regression analysis, each unitary increase of miR-9 expression was associated with worse OS (HR: 1.48; 95%CI: 1.06–2.09; p = 0.023). Kaplan Meier plot also shows worse prognosis in the breast cancer subgroup with values above the median as compared to those below the median (Fig. 2c). This association was confirmed in a multivariable Cox regression model including age at diagnosis, tumour size and lymph node status (HR: 1.57; 95%CI: 1.08–2.29; p = 0.018). Nevertheless, no statistical significant association was detected in a model including ER and PgR status together with age, tumour size and lymph node status (HR: 1.38; 95%CI: 0.91–2.1; p = 0.12). These results, however, need to be interpreted cautiously due to the short follow-up time of the patients’ cohort (Median: 19 months; IQR: 9–45 months). Survival analysis within the PAM50 molecular subgroups could not be performed due to the low number of events (n = 20) in the patients’ cohort.

### Evaluation of miR-9 expression regulation by epigenetic mechanisms

**Figure 3:**
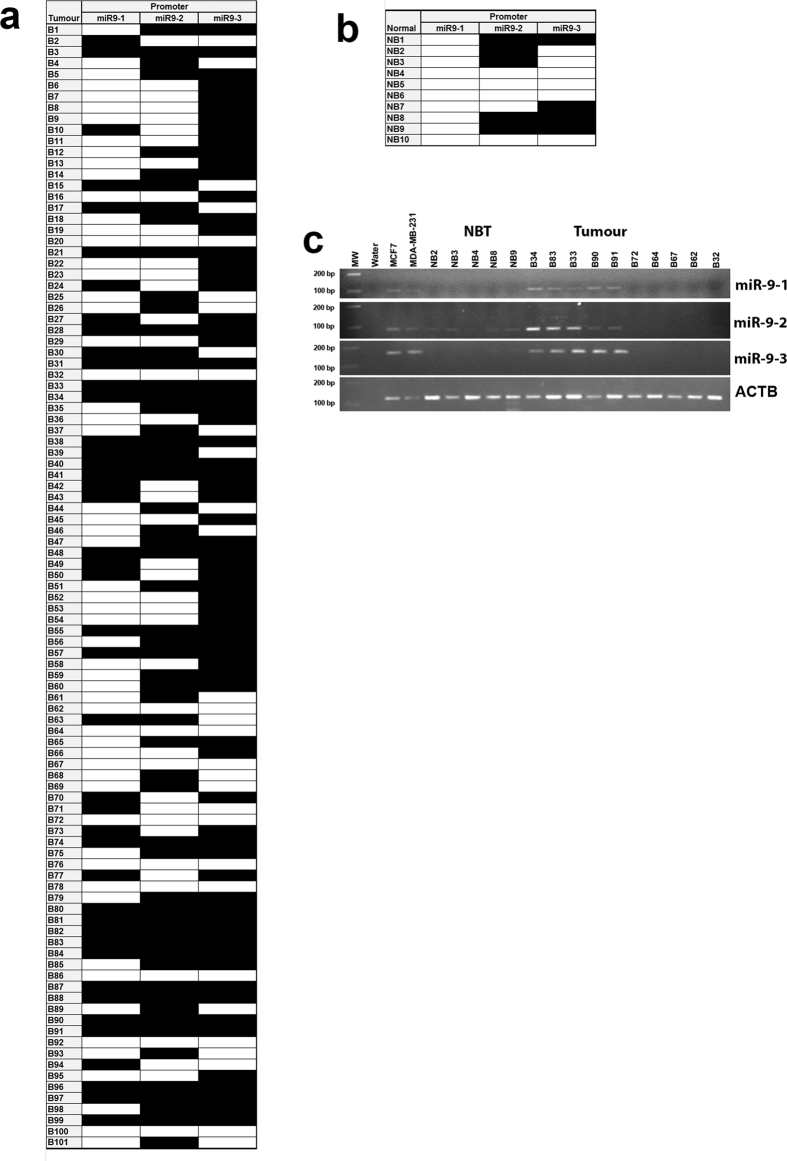
Methylation analysis by MSP at the three genomic loci (*MIR9-1, MIR9-2* and *MIR9-3*) encoding for miR-9-5p. Methylation distribution in (**a**) tumours (n = 101) and (**b**) normal breast tissues (n = 10); *black box* methylated sample; *white box* unmethylated sample. (**c**) Representative results of MSP analysis for *MIR9-1, MIR9-2* and *MIR9-3* promoter regions in normal breast tissues (NBT) and tumours. A region of *ACTB* gene not containing CpGs was used to evaluate bisulphite conversion. MW, molecular weight marker; Water PCR negative control; MCF7 and MDA-MB231 methylation positive controls.

The methylation status of the three *MIR9* promoters (*MIR9-1, MIR9-2, MIR9-3*) was analyzed by Methylation Specific PCR (MSP) using the primer sets designed by Lujambo *et al*.^7^ mapping to the CpG region encompassing the *MIR9* genes transcription start sites validated by RACE. The analysis could be performed in 101 of 131 breast cancer cases and 10 out of 12 epithelial normal breast tissues (NBTs) due to the lack of genomic DNA for the remaining samples. Overall, methylation in at least one of the three promoters was detected in 6 out of 10 NBTs (60.0%) and in 90 out of 101 tumours (81.1%) (p = 0.029) (Fig. 3a,b). A statistically significant difference in methylation frequency between normal and tumour samples was detected only for the *MIR9-1* promoter (NBTs 0/10, Tumour 43/101, p = 0.006). The *MIR9-2* promoter was methylated in 5 out of 10 NBTs and 58 out of 101 tumours (p = 0.744) and *MIR9-3* promoter in 4 out of 10 NBT and in 71 out of 101 tumours (p = 0.075) (Table S4). Although not statistically significant, a slight trend of methylation frequency was found only when the combined status at the three promoters was evaluated (p = 0.098) (Table S5).

Next, we moved forward to evaluate the expression of the three pri-miRNAs encoded by *MIR9* genes in 90 out of the 101 breast cancer cases and 9 out of the 12 epithelial normal breast tissues analysed for methylation. For all three pri-miRNAs, the expression levels was significantly lower in tumour samples as compared with NBTs (Table S6). Furthermore, when we investigated the association between each pri-miRNA expression and promoter methylation status at the correspondent *MIR9* locus, no statistically significant differences were detected between unmethylated and methylated samples ether in tumours (Table S7) or normal tissues (Table S8) for any of the pri-miRNAs.

### Ingenuity Pathway Analysis (IPA) highlighted the hormone receptors-mediated role of miR-9-5p in the pathogenesis of breast cancer

**Figure 4:**
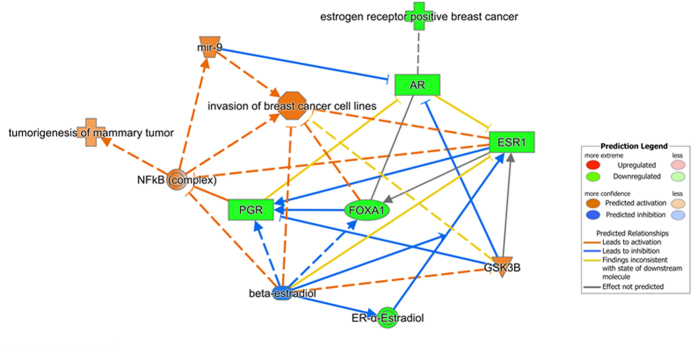
Ingenuity Pathway analysis (IPA) highlights the pivotal role of hsa-miR-9-5p in hormone regulated pathways leading to breast cancer tumorigenesis. miR-9-5p negatively regulates the androgen receptor (AR) and oestrogen receptor (ER). Hormone receptor downstream effects are mediated by the NF-kB complex that, in turn, is likely to induce miR-9-5p expression.

Differential expression (DE) analysis was carried out after dichotomizing the samples profiled in the TCGA dataset on the basis of the miR-9-5p median expression value: cases with miR-9-5p expression below −0.1679 were classified as miR-9^low^, while cases with miR-9-5p above −0.16 as miR-9^high^. DE analyses were carried out through one-way ANOVA. *In-silico* functional enrichment analysis was performed on DE mRNAs having a significant (p < 0.05) absolute Fold Change greater than 2. Then, we obtained a signalling network involving miR-9, ESR1 and PGR, together with upstream regulators, other molecules and interesting functions. Results from this analysis highlighted the pivotal role of miR-9-5p (Fig. 4) in breast cancer tumourigenesis and invasion through the negative regulation of the androgen receptor (AR) and oestrogen receptor (ER). In this model, their downstream effects are mediated by the NF-kB complex that, in turn, is likely to induce the expression of miR-9-5p. Beta-estradiol is an upstream regulator (z-score = −1.529, p-value = 1.87e-16), and inhibits ESR1, which in turn interacts with PGR. Since PGR is known to inhibit the NF-kB complex, its actual inactive state contributes to the increased activity of NF-kB. Perturbations of this complex regulatory loop may likely culminate in the aberrant upregulation of miR-9-5p and thus to breast cancer tumourigenesis and invasion. Interestingly, our target prediction analysis indicates both ESR1 and AR mRNAs as targets of miR-9-5p, although experimental evidences have been reported only for ESR1 (PMID: 23824327)^30^,^31^,^32^,^33^.

#### Evaluation of AR, FOXA1 and GSKB3 expression in the discovery set

To validate the IPA model, we measured the expression of AR, FOXA1 and GSKB3 at mRNA level in 122 out of the 131 breast cancer cases. A significant positive correlation was found between the percentage of ER and PgR expressing cells and AR (r = 0.31, p < 0.001 and r = 0.34 p < 0.001, respectively) and FOXA1 (r = 0.40 p < 0.001 and 0.41, p < 0.001, respectively) mRNA levels, the latter being also negatively correlated with miR-9-5p expression (r = −0.29; p = 0.001).

### Search for Oestrogen Responsive Elements (ERE) in the promoter regions of MIR9 genes

To explore whether ER may directly regulate the expression of any of the *MIR9* genes, we implemented a computational strategy to find putative or experimentally validated regulatory elements in the 3kbp-spanning regions upstream the genes. By using Multiple EM for Motif Elicitation (*MEME*) method, we found a pattern located within the *MIR9-1* upstream region (significance of motif occurrence: p-value = 9.5 × 10^−5^, q-value = 0.27), in the region chr1:156387407–156387423, strand “ + ” (i.e., the same of the gene). This region was investigated also by means of the *RSAT matrix-scan* web-tool, which returned also the complementary sequence (weights = 0.3 and 1.5; p-values = 1.00E-4 and 6.5E-5, respectively, for the two elements). One ERE motif was found by both methods in the upstream region of *MIR9*-3 at the locus with coordinates: chr15:89908739–89908755, with the same strand orientation of the gene (MEME p-value = 5.93E-5, q-value: 0.15; RSAT weight and p-value: −1.9 and 2.4E-4). We also found a third motif within the region chr1:156388941–156388957 (*MIR9-1* upstream region) by both MEME and RSAT (MEME p-value = 4.57E-5, q-value = 0.26; RSAT weight = −0.7, p-value = 1.5E-4), although located in the opposite strand to the gene. For what concerns the regulation of *MIR9-2*, a relevant motif was found by both tools in the region chr5:87962865–87962881, although located in the opposite strand, with respect to the miRNA gene (MEME p-value = 1.54E-5, q-value = 0.046; RSAT weight and p-value = 6.72 and 6.91E-6, respectively).

We also sought for predicted Transcription Factor Binding Sites in public collections, in particular in the “tfbsConsSites” UCSC track, and found a putative ESR1 binding site (score = 1.97, corresponding to a p-value < 0.05) within the *MIR9-2* upstream region, even if in the opposite strand. Thus, regulatory mechanisms look quite complex for what concerns the *MIR9-2* gene, with potential responsive elements located with an opposite orientation with respect to it and probably related to the *LINC00461* gene, which is transcribed within the same genomic region. A summary of the detected regions is provided in Table 2.

## Discussion

In our work, we sought to determine the biological as well as clinical relevance of miR-9 in breast cancer pathology by analyzing different levels of gene expression regulation, reconstructing the network of interactions miR-9 holds within the cell context and evaluating the correlation with patients’ prognosis.

At first, we evaluated the expression of both mature transcripts, miR-9-5p and miR-9-3p, in a cohort of 131 cases with at least 5-years follow-up and 12 control tissues from reductive mammoplasty. This analysis demonstrated a significant upregulation of miR-9-5p and miR-9-3p in tumours as compared with normal tissues. Although the expression of the two miRNAs was overall coordinated, a high variability among samples was observed. Investigating further, it was clear that this variability is strongly related to the expression of the oestrogen receptor. Indeed, the only correlation with clinicopathological parameters for miR-9-5p was a strong negative correlation with percentage of cells positive for both ER and PgR by IHC examination. By contrast, miR-9-3p expression negatively correlated only with ER expression, patient’s age and tumour dimension, and positively correlated with ki67/miB1 expression. These results, and in particular the correlation with PgR expression, suggest that while miR-9-5p is directly involved in oestrogen regulated pathways, the association of miR-9-3p with ER is mainly due to the coordinate expression with miR-9-5p.

The fact that both miR-9 mature transcripts may play different roles in breast carcinogenesis is further demonstrated by the differential expression in the breast cancer subgroups identified by the surrogate molecular classification showing a significant upregulation of miR-9-5p in both triple negative and HER2 amplified breast cancers as compared with luminal subtypes, but a miR-9-3p up-regulation limited to triple negative tumours as compared with luminal and HER2 amplified tumours. The analysis of the TCGA Breast Cancer dataset demonstrated also a statistical significant difference between the two luminal subtypes and triple negative and HER2-amplified BCs, suggesting that miR-9 expression might be used to refine the breast cancer molecular classification.

miR-9 has also been identified as an epigenetically regulated miRNA in several tumour types^7^,^8^,^13^, although inconsistent, even opposite, data about the correlation between *MIR9* promoters methylation status and expression have often been reported^7^,^8^,^9^,^10^,^11^,^12^,^13^. According to the largest collection of normal tissues (GTEx), miR-9 is highly expressed only in the brain as demonstrated by both RT-qPCR and LNA-ISH analyses^34^. Furthermore, miR-9 has been found highly variable across different carcinomas 11,15,16,17,18,19,20, although results from a recent meta-analysis support that its overexpression rather than down regulation is associated with worse prognosis^21^.

In light of this, we decided to clarify the role of methylation in regulating the expression of *MIR9* loci by assessing the methylation status of the three *MIR9* promoter regions and the expression levels of the primary transcripts (i.e. pri-miR-9-1, pri-miR-9-2, and pri-miR-9-3) in our cohort of breast cancers and normal tissues. We found that gene promoter methylation was more frequent in tumours than in normal breasts, although the expression levels of all the pri-miRs were lower in tumours as compared with normal breast tissues. The correlation analysis between the methylation status at the three *MIR9* promoter regions and expression levels of both pri-miRs and mature miR-9-5p and miR-9-3p failed to find a significant correlation for any of the transcripts either in tumours or normal breasts. At this point, the lack of association between the high rate of methylation at the *MIR9* promoters and upregulation of miR-9-5p and miR-9-3p in breast tumours might suggest that additional layers of complexity in the regulation of miR-9 expression do exist and may include both a multifactorial transcriptional control and pri-miRNA processing regulatory mechanisms that are normally quiescent in physiological conditions. For instance, it is well known that DNA methylation is one of the hallmarks associated with cancer^35^,^36^, but while these epigenetic mechanisms were initially believed to confer stable, long-term gene silencing, now it is clear that this modification is reversible^37^,^38^. In this context, Hsu *et al*.^13^ found that the combination of 5-Azacytidine with trichostatin was necessary to obtain an increased miR-9-5p expression in breast cancer cell lines bearing methylation at the *MIR9* promoters, implying that chromatin remodelling plays a pivotal role in gene expression control as well.

Therefore, with the aim to uncover further regulators of miR-9 expression, we investigated the biological significance of the inverse association between miR-9-5p and ER and PgR by searching for the putative interactions among miR-9-5p, ER and PgR and their upstream and downstream regulators through IPA. In addition, we also searched for oestrogen response elements (ERE) in the upstream region of *MIR9* genes which may represent another putative mechanism of transcriptional regulation. Overall, in agreement with previous reports^13^,^32^,^39^, the IPA built a regulatory loop by which miR-9-5p expression is influenced by steroid hormone receptors, involving AR, FOXA1 and GSKB proteins, and leading to its activation through the NF-kB complex. On the other side, miR-9-5p had been experimentally demonstrated to directly target ESR1 mRNA^32^, thus implying a negative feedback mechanism of intracellular regulation. Finally, the identification of putative ER binding sites within *MIR9* promoters rises the possibility that ER may directly affect the *MIR9* transcription. Consistently with this model, AR and FOXA1 mRNA expression positively correlated with percentage of ER and PgR expressing cells in our cohort of breast tumours, but only FOXA1 mRNA expression negatively correlated with miR-9-5p expression. We might assume that the absence of a significant inverse correlation between miR-9-5p and AR, which is predicted to be its direct target, is due to the limited number of highly miR-9-5p expressing Triple Negative and HER2 amplified cases in our discovery cohort.

However, the inverse correlation between FOXA1 and miR-9-5p might help unravel an intricate mechanism of cooperation between epigenetic and genetic mechanisms of *MIR9* expression control. Indeed, it has recently been demonstrated that the members of the FOXA family may localize at distal regulatory enhancers to establish chromatin competency for subsequent recruitment of collaborating transcription factors. Among these, FOXA1 has been demonstrated to cooperate with sex hormone receptors, including estrogen and androgen receptors (ER and AR) and influence ER binding to chromatin^40^,^41^. This would explain the positive correlation we found with ER. Also, enhanceosome comprising ER, FOXA1, and GATA3 was found to be sufficient to restore estrogen responsiveness in ER− breast cancer cells^42^. Even more interesting, depletion of FOXA1 produced localized reestablishment of a methylation pattern across FOXA1-bound regions, mostly represented by active promoters and enhancers, whereas overexpression of FOXA1 commits its binding sites to active DNA demethylation which resulted to be tightly coupled with estrogen receptor genomic targeting and estrogen responsiveness^41^. Altogether, these data not only support the reliability of our IPA analysis but suggest a putative complex cooperation among methylation at the three *MIR9* promoters, chromatin remodelling factors and transcriptional regulators including the NF-kB complex and FOXA1, which fine tune miR-9-5p expression levels in both tumour and normal epithelial components. According to IPA model, these mechanisms would be under the control of estradiol through the regulation of *ESR1* expression. Any factor perturbing this regulatory loop may favour cancer development and/or affect progression. We may speculate that in receptor positive breast cancer, the down regulation of NF-kB complex induced by PgR, with the possible contribution of FOXA1 would lower miR-9-5p expression, and thus increase translation of the ESR1 mRNA. In receptor negative breast tumours, the mechanism would be reversed: the absence of ER would lead to a reduced PgR expression^43^,^44^, and allow NF-kB complex activation and miR-9-5p upregulation that in turn might contribute to the maintenance of ER negative status by negatively targeting ESR1 mRNA and promote the development of typical features associated with clinical aggressiveness of breast cancer.

To determine whether miR-9-5p might represent a useful molecular biomarker, we evaluated the correlation between its expression and survival in our discovery cohort and in the breast cancer TCGA dataset. Although in the TCGA dataset we could only evaluate the overall survival, univariable analyses suggested that higher levels of miR-9-5p were associated with worse patient’s prognosis, although this effect was strongly dependent on the relationship between miR-9-5p and hormone receptor status. In our discovery cohort, we did not find any association with survival. However, in the subgroup of ER positive patients, both univariable and multivariable Cox regression analyses suggested that miR-9-5p (but not miR-9-3p expression) was associated with all time-to-event outcomes. Thus, if we consider the biological interactions described above, we cannot rule out that miR-9-5p expression may have a differential predictive value according to breast cancer molecular subtypes, and may affect response to treatments. We may hypothesize that ER positive tumours expressing high level of miR-9-5p may exhibit a different response to anti-oestrogen treatments as compared with low expressing tumours. On the other side, since miR-9-5p expression seems to be related to invasive and metastatic propensity of cancer cells, the use of miRNA inhibitors in ER negative breast cancer expressing high levels of miR-9-5p may revert this aggressive phenotype and enhance the expression of hormone receptors, inhibiting pathways associated with metastatic behaviour and even rendering patients responsive to hormonal therapies.

## Conclusions

In conclusion, our results indicate that miR-9-5p up-regulation in breast cancer is the result of a complex interplay between epigenetic mechanisms and oestrogen regulated pathways. The evaluation of miR-9-5p expression in primary breast cancer might help the identification of breast cancer molecular subgroups characterized by different clinical behaviour and represent a target for novel therapeutic interventions aimed to re-induce hormone receptor expression in ER negative breast cancers.

## Methods

### Study Design

In this study, we evaluated hsa-miR-9-5p and hsa-miR-9-3p expression in a discovery cohort of 131 breast cancer cases with 5-years follow-up. The study was conducted according to the REporting of tumour MARKer Studies (REMARK) guidelines^45^ and a prospectively written research, pathologic evaluation, and statistical analysis plan. Breast cancer tissues were collected at the Breast-Unit, IRCCS “Casa Sollievo della Sofferenza”. Upon receipt from surgery, tissue from the bulk of the tumour was snap-frozen in liquid nitrogen and stored at −80 °C until used. In order to be included in the study, patients must be female, aged more than 18 years, and tumour must be more than 1.0 cm in diameter due to legal reasons.

All methods were carried out in accordance with international (Helsinki Declaration 7^th^ rev, 2013, EU Directive 2004/23/EC) and Italian (D. Lgs. 30/06/2003, n. 196) regulations for research on human subjects. All experimental protocols were approved by the Ethical Committee of the IRCCS “Casa Sollievo della Sofferenza” (Prot N 140/CE). Prior written and informed consent was obtained from all patients in accordance with the experimental protocol approved by the Ethical Committee.

### Clinicopathological Data

Pathological assessment includes evaluation of histological type, grade and stage. Oestrogen Receptor (ER), Progesterone Receptor (PgR), Ki-67 labelling index and HER2 status^46^,^47^. Clinical data were collected at each of the scheduled follow up times.

### DNA and RNA extraction from tissues

5-μm-thick sections were cut from OCT embedded frozen specimens and H&E stained for visual inspection under the microscope to ensure that tumour samples contained at least 70% cancer cells, and to confirm that tissues obtained from reductive mammoplasty contained normal epithelial gland component. DNA was extracted from one half of frozen sections as described previously^48^. Total RNA was extracted from the second half of the fresh frozen samples using the TRIzol reagent according to the manufacturer’s instructions. RNA quality was evaluated by using 2100 Expert Analyzer (Agilent Technology) and only RNAs with RNA Integrity Number (RIN) ≥ 7.0 were processed. RNA concentration was quantified by the absorbance measurement at 260 and 280 nm using the NanoDropTM.1000 spectrophotometer (NanoDrop Technologies).

### Quantitative Reverse Transcription Polymerase Chain Reaction (RT-qPCR) analysis

For miRNAs analysis, single-stranded cDNA was synthesized from 5.5 ng of total RNA by using TaqMan MicroRNA Reverse Transcription Kit (Thermo Fisher Sc) and 50 nM specific stem-loop RT primers (hsa-miR-9-5p ID 000583, hsa-miR-9-3p ID 002231, and RNU48 ID 001006), according to manufacturer’s instructions. Total cDNA was synthesize from 400ng of total RNA by using SuperScript™ III First-Strand Synthesis SuperMix (Thermo Fisher Sc) with random hexamers according to manufacturer’s instructions.

A relative quantification method with standard curve was developed to determine miRNA and gene expression in tissues^48^. MiRNA expression was assessed by TaqMan microRNA assay, and the expression of mRNAs was measured by TaqMan probes. TaqMan microRNA assays were as follows: hsa-miR-9-5p (ID 000583), hsa-miR-9-3p (ID 002231), TaqMan gene expression probes were as follows: pri-miR-9-1 (ID Hs03303201_pri), pri-miR-9-2 (ID Hs03303202_pri), pri-miR-9-3 (ID Hs03293595_pri), AR (ID Hs00171172_m1), FOXA1 (ID Hs04187555_m1), GSK3B (ID Hs04187555_m1). miRNA expression was normalized to that of RNU48 (ID 001006), while gene expression analysi, was normalized to that of *RPLPO* (ID Hs04187555_m1).

Real-time PCR reactions were performed in 384-well plates on ABI PRISM 7900HT Sequence Detection System (Life Technologies). All samples were run in triplicates. The analysis was performed by using SDS 2.4 software (Thermo Fisher Sc). For each miRNA, gene and reference genes, standard curves were constructed by plotting the threshold cycle (Ct) values against logarithm10 of the copy number and fitting by linear least square regression. The level of miRNA and gene expression in each sample was determined as the ratio of the miRNA/gene copy number to the reference copy number and then multiplied by 1000 for easier tabulation (e.g. hsa-miR-9-5p/RNU48) x 1000). Efficiency of amplification was calculated for each real-time PCR run for miRNAs, genes and reference genes as follows: E = (10^(−1/slope)-1) using the slope of the standard curve plots of Ct versus log input of cDNA. (Table S9).

### Methylation specific PCR (MSP)

DNA samples were subjected to bisulphite conversion and DNA purification by using the Epitect Bisulfite kit (Qiagen Sci) according to manufacturer’s instruction. An MSP reaction for the *ACTB* gene promoter region not containing CpGs was performed as control of the bisulphite conversion for each sample^49^. Forward and reverse primers for the bisulphite-converted methylated sequences of *MIR9-1, MIR9-2* and *MIR9-3* promoter regions were those described previously by Lujambio *et al*.^7^. A stepdown-PCR (SD-PCR) approach was set up and conditions were as follows: *ACTB* region: 94 °C 30”−64 °C 30”−72 °C-30” for 5 cycles, 94 °C 30”−62 °C 30”−72 °C-30” for 5 cycles, 94 °C 30”−60 °C 30”−72 °C-30” for 25 cycles. *MIR9* gene promoters: 94 °C 30”−62 °C 30”−72 °C-30” for 5 cycles, 94 °C 30”−60 °C 30”−72 °C-30” for 5 cycles, 94 °C 30”−58 °C 30”−72 °C-30” for 25 cycles. Expected amplicons size were 133 bp for *ACTB*, 103 bp for *MIR9-1*, 92 bp for *MIR9-2* and 162 bp for *MIR9-3*. CpGenome Universal Methylated DNA (Serologicals Corp., Norcross, GA) and MCF7 and MDA-MB-231 cell lines were used as positive controls. PCR products were run on a 3% Agarose gel, stained with Midori Green (Nippon Genetics Europe GmbH) and visualized under UV light.

### Search for regulatory elements in *MIR9*

The computational strategy used to find regulatory elements in the vicinity of *MIR9* genes was based on a double approach. Primarily, we used two motif analysis web-suites (MEME)^50^ and RSAT^51^ to eventually map the ESR1 responsive element (ERE) to the 3kbp-spanning regions upstream to the three *MIR9* genes. A position-specific scoring matrix (PSSM) for ER was retrieved from JASPAR^52^, matrix ID: MA0112.3) and provided in input to the MEME-FIMO and RSAT “matrix-scan” tools. GRCh37 genomic sequences were extracted from ENSEMBL release 85^53^. Both tools were run with default settings. Significant MEME motifs usually exhibit low p-values and q-values (indicating non-randomness of the found motifs), while RSAT significant results have positive (or around 0) weights and low p-values.

In addition, we verified if predicted or validated ERE binding sites were already reported by public datasets. We used the UCSC Data Integrator tool^54^ to detect functional or predicted elements that overlap with the considered regions. In particular, we selected the “tfbsConsSites”, “wgEncodeRegTfbsClusteredV3”, “-Rep2” and “wgEncodeHaibTfbsEcc1EralphaaV0416102Est10nm1hPkRep1 subtracks from the UCSC “Regulation” main track. The first track reports evolutionary conserved binding sites for a large set of transcription factors; the second one synthesizes the results of a vast series of ENCODE^55^ Sequencing Projects, by clustering and normalizing peak signals on different ENCODE cell contexts. The third and fourth tracks define a subset of the ENCODE collection where ESR1 (or “ER”, “oestrogen receptor”, Ensembl Gene accession number: ENSG00000091831) was reported as target factor (read more at https://www.encodeproject.org/experiments/ENCSR000BQR/).

### Statistical Analysis

Patients’ baseline characteristics were reported as median along with interquartile range (IQR, i.e. first-third quartiles) or frequencies and percentages for continuous and categorical variables, respectively.

The assumption of normality distribution was checked by means of Q-Q plots and Shapiro-Wilks test. As the distribution of miRs expression levels (i.e. miR-9-5p and miR-9-3p) in the discovery set was log-normal, all statistical analyses which involved the miRs expression in the discovery set were performed on their log transformed values. Nonparametric statistics were instead used for TCGA dataset. Comparisons between miRs levels and clinical pathological characteristics were assessed by Pearson correlation coefficient and two-sample t-test (or ANOVA model as appropriate) for continuous and categorical variables in the discovery set, respectively. The association of methylation distribution at the three *MIR9* promoter regions between tumours and normal breast tissues was assessed using Fisher exact test. In the TCGA dataset, to test correlations among miR-9, ESR1 and PGR mRNAs Spearman r correlation coefficients were estimated. Kruskall-Wallis and Mann-Whitney tests were used to evaluate miR-9 differential expression among ER positive/negative samples, PR positive/negative samples and PAM50 subtypes. Differentially expressed genes between miR-9^high^ and miR-9^low^ groups were identified by the implementation of the one-way ANOVA of Partek^®^ Genomics Suite^®^ software, version 6.6. Pathway analysis and the final network were generated by QIAGEN’s Ingenuity^®^ Pathway Analysis (IPA^®^, QIAGEN Redwood City, www.qiagen.com/ingenuity).

Time-to-event analysis was performed in patients without metastases at diagnosis by univariable and multivariable proportional hazards Cox regression models. Multivariable Cox models included: the exposure of interest (i.e. miRs expression) and age at diagnosis, presence of lymph nodes, positivity of estrogen receptor, positivity in progesterone, the presence of neoadjuvant therapy as further covariates. Risks were reported as Hazard Ratios (HR) along with their 95% Confidence Interval (95%CI). miRs expressions were treated both as continuous variables (i.e. HRs were expressed for each unitary increase in expression levels) and were also categorized with respect to median and tertiles. In the latter case, tertiles identifies low, intermediate and high expression group, respectively. Overall Survival (OS) was defined as the time between the enrollment date and cancer related death. Progression Free Survival (PFS) was defined as the time between the enrollment date and the tumour progression. Metastasis Free Survival (MFS) was defined as the time between the enrollment date and the development of distant metastases. Mortality and disease progression rates were reported as number of events per 100 person-years. Survival curves were drawn using Kaplan-Meier method. A two-sided *p*-value < 0.05 was considered for statistical significance.

All statistical analyses were performed using SAS Release 9.4 (SAS Institute, Cary, NC, USA). Plots were produced using Comprehensive R Archive Network (CRAN) version 3.3.1 (packages: *stats, survival*).

## Additional Information

**How to cite this article:** Barbano, R. *et al*. Stepwise analysis of *MIR9* loci identifies miR-9-5p to be involved in Oestrogen regulated pathways in breast cancer patients. *Sci. Rep.* **7**, 45283; doi: 10.1038/srep45283 (2017).

**Publisher's note:** Springer Nature remains neutral with regard to jurisdictional claims in published maps and institutional affiliations.

## Supplementary Material

Supplementary Tables

## Figures and Tables

**Table 1:** miR-9-5p and miR-9-3p expression in the breast cancer subgroups identified by surrogate molecular classification.

BC subgroups	miR-9-5p			miR-9-3p		
	n	Median (IQR)	p-value*	n	Median (IQR)	p-value*
Luminal	83	0.17 (0.07–0.40)	0.008	76	0.28 (0.14–0.67)	0.004
Triple negative	23	0.28 (0.16–3.54)		22	1.26 (0.36–2.59)	
HER2-amplified	18	0.44 (0.22–0.58)		17	0.24 (0.13–0.96)	

*p-value from ANOVA models on log-transformed miRs expression levels.

**Table 2:** Detected ERE motifs within the 3 kbp long upstream regions.

	chr	start	end	strand	RefSeq ID	Results (resource/region/sequence)
miR-9-1	1	156390123	156390230	+	ENST0000038519, NR_029691	“meme” and “rsat”:1:156388941–156388957:−1; CTGGACAAAATGACCTT.
						“meme” and “rsat”:1:156387407–156387423:1; AGTGTCACCGTGATGTC.
miR-9-2	5	87962671	87962757	−	ENST0000038483, NR_030741	“tfbsConsSites”:V$ER_Q6, 5:87962863–87962882:1, TTTATGTAAATATGACCTGG.
						“meme” and “rsat”: 5:87962865–87962881:1, TATGTAAATATGACCTG.
miR-9-3	15	89911248	89911337	+	ENST0000038508, NR_029692	“meme” and “rsat”: 15:89908739–89908755:1;CTGGTCAGCCTCACCTG.

Last column reports resource name (“meme”, “rsat” and “tfbsConsSites” for MEME-FIMO, RSAT matrix-scan and UCSC Data Integrator Tool, respectively), motif chromosomal coordinates and sequence.
