# Supplementary material for: Stepwise analysis of MIR9 loci identifies miR-9-5p to be involved in Oestrogen regulated pathways in breast cancer patients
Source: Sci Rep. 2017 Mar 27;7:45283. doi: 10.1038/srep45283 (PMC5366901; doi:10.1038/srep45283)
Supplement: Supplementary Tables [file srep45283-s1.doc]

**Stepwise analysis of *MIR9* loci identifies miR-9-5p to be involved in Oestrogen regulated pathways in breast cancer patients.**

Raffaela Barbano^1^**^ϯ^**, Barbara Pasculli^1^**^ϯ^**, Michelina Rendina^1^, Andrea Fontana^2^, Caterina Fusilli^3^, Massimiliano Copetti^2^, Stefano Castellana^3^, Vanna Maria Valori^4^, Maria Morritti^4^, Paolo Graziano^5^, Ciuffreda Luigi^6^, Michelina Coco^1^, Francesco Picardo^7^, Tommaso Mazza^3^, Ella Evron^8^, Roberto Murgo^6^, Evaristo Maiello^4^, Manel Esteller^9,10,11^, Vito Michele Fazio^1,7^, Paola Parrella^1*^

^1^Laboratory of Oncology, ^2^Unit of Biostatistics, ^3^Unit of Bioinformatics, ^4^Oncology Department, ^5^Pathology Department, ^6^Breast Unit, IRCCS Casa Sollievo della Sofferenza, San Giovanni Rotondo, Italy; ^7^Laboratory of Molecular Medicine and Biotechnology, Center of Integrated Research, Campus Bio-Medico University of Rome; ^8^Assaf Harofeh Medical Center Zerifin, Affiliated with Tel Aviv University, Sakler School of Medicine, Israel; **^9^**Cancer Epigenetics and Biology Program (PEBC), Bellvitge Biomedical Research Institute (IDIBELL), L’Hospitalet de Llobregat, Barcelona, Catalonia, Spain; ^10^Institució Catalana de Recerca i Estudis Avançats (ICREA), Barcelona, Catalonia, Spain,^11^Department of Physiological Sciences II, School of Medicine, University of Barcelona, Barcelona, Catalonia, Spain

***Corresponding Author:**

Paola Parrella, MD

Laboratory of Oncology,

IRCCS Casa Sollievo della Sofferenza

San Giovanni Rotondo (FG)

71013

Tel +390882416261

Fax +390882416264

e-mail [pparrella@operapadrepio.it](mailto:pparrella@operapadrepio.it)

^ϯ^ **These authors contributed equally to this work.**

**Supplemental Table 1. Clinico-pathological characteristics of the discovery cohort.**

| **Variable** | **Category** | **All (N=131)** |
| --- | --- | --- |
| **Age at diagnosis** | Median (IQR) | 59.77 (46.54-71.19) |
| **Menopause – N(%)** | No | 34 (25.95) |
|  | Yes | 97 (74.05) |
| **Site - N(%)** | Right | 66 (50.38) |
|  | Left | 63 (48.09) |
|  | Bilateral | 2 (1.53) |
| **Tumor history - N(%)** | Primary | 120 (91.60) |
|  | Relapse | 11 (8,39) |
| **Surgery type - N(%)** | Mastectomy | 95 (72.51) |
|  | Quadrantectomy | 36 (27.48) |
| **Tumor histotype - N(%)** | Invasive ductal | 121 (92.36) |
|  | Invasive lobular | 10 (7.63) |
| **Tumor size (cm)** | Median (IQR) | 2.5 (2.0-3.5) |
| **Tumor - N(%)** | Missing values | 2 |
|  | T1 | 35 (27.0) |
|  | T2 | 58 (45.2) |
|  | T3 | 6 (4.8) |
|  | T4 | 30 (23.0) |
| **Lymph nodes - N(%)** | Missing values | 1 |
|  | N0 | 48 (36.92) |
|  | N1 | 82 (63.07) |
| **Metastases - N(%)** | Absent | 122 (93.13) |
|  | Present | 9 (6.87) |
| **Ki67/mib1** | Median (IQR) | 30 (20-50) |
| **Grading – N(%)** | Missing values | 15 |
|  | 1 | 12 (10.34) |
|  | 2 | 55 (47.41) |
|  | 3 | 49 ( 42.24) |
| **Stage - N(%)** | Missing values | 2 |
|  | I | 20 (15.55) |
|  | II | 58 (44.96) |
|  | III | 42 (32.55) |
|  | IV | 9 (6.97) |
| **Estrogen Receptor** | Median (IQR) | 60 (0-90) |
|  | Positive – N (%) | 83 (63.35) |
| **Progesterone Receptor** | Median (IQR) | 8 (0-70) |
|  | Positive – N (%) | 66 (50.38) |
| **HER2neu - N(%)** | Missing values | 7 |
|  | No | 90 (72.58) |
|  | Yes | 34 (27.42) |
| **Surrogate Molecular classification - N(%)** | Missing values | 7 |
|  | Luminal | 83 (66.94) |
|  | Triple Negative | 23 (18.55) |
|  | HER2 | 18 (14.52) |
| **Overall survival time (months)** | Median (IQR) | 67.03 (38.17-88.07) |
| **Progression Free Survival (months)** | Median (IQR) | 61.17 (26.07-82.53) |
| **Metastases Free Survival (months)** | Median (IQR) | 61.17 (29.03-83.07) |

IQR: Interquartile range (i.e. first-third quartiles)

**Supplemental Table 2.** Cox regression models evaluating the association between miR-9-5p and miR-9-3p expression and Overall Survival (OS), Progression Free Survival (PFS) and Metastases Free Survival (MFS) among non metastatic patients of the discovery set, within the luminal breast cancer subgroup (N=76).

| **Model** | **Outcome** | **miRNA** | **Tertile group^** | **HR (95%CI)** | **p-value** |
| --- | --- | --- | --- | --- | --- |
| Univariable  Analysis | OS | hsa miR-9-5p | **II vs. I** | **4.22 (1.14-15.62)** | **0.031** |
|  |  |  | III vs. I | 1.10 (0.22-5.48) | 0.904 |
|  |  | hsa miR-9-3p | II vs. I | 0.73 (0.23-2.32) | 0.598 |
|  |  |  | III vs. I | 0.35 (0.09-1.37) | 0.134 |
|  | PFS | hsa miR-9-5p | **II vs. I** | **3.20 (1.10-9.25)** | **0.032** |
|  |  |  | III vs. I | 0.62 (0.15-2.62) | 0.520 |
|  |  | hsa miR-9-3p | II vs. I | 0.93 (0.34-2.58) | 0.893 |
|  |  |  | III vs. I | 0.44 (0.13-1.46) | 0.180 |
|  | MFS | hsa miR-9-5p | **II vs. I** | **3.22 (1.11-9.33)** | **0.031** |
|  |  |  | III vs. I | 0.64 (0.15-2.67) | 0.537 |
|  |  | hsa miR-9-3p | II vs. I | 0.90 (0.33-2.50) | 0.840 |
|  |  |  | III vs. I | 0.44 (0.13-1.45) | 0.176 |
| Multivariable Analysis* | OS | hsa miR-9-5p | **II vs. I** | **9.64 (1.18-79.02)** | **0.035** |
|  |  |  | III vs. I | 2.26 (0.22-22.75) | 0.490 |
|  |  | hsa miR-9-3p | II vs. I | 0.63 (0.17-2.34) | 0.494 |
|  |  |  | III vs. I | 0.25 (0.05-1.26) | 0.092 |
|  | PFS | hsa miR-9-5p | **II vs. I** | **6.47 (1.38-30.31)** | **0.018** |
|  |  |  | III vs. I | 1.08 (0.17-6.87) | 0.931 |
|  |  | hsa miR-9-3p | II vs. I | 1.01 (0.31-3.31) | 0.983 |
|  |  |  | III vs. I | 0.31 (0.07-1.29) | 0.107 |
|  | MFS | hsa miR-9-5p | **II vs. I** | **6.51 (1.39-30.54)** | **0.018** |
|  |  |  | III vs. I | 1.11 (0.18-7.05) | 0.910 |
|  |  | hsa miR-9-3p | II vs. I | 0.97 (0.30-3.14) | 0.954 |
|  |  |  | III vs. I | 0.31 (0.07-1.29) | 0.107 |

*Cox models were adjusted for: age at diagnosis, presence of lymph nodes, positivity of estrogen receptor, positivity in progesterone, the presence of neoadjuvant therapy; ^ tertiles groups (i.e. I, II, III) were referred to low, intermediate and high expression, respectively.

**Supplemental Table 3. Clinico-pathological characteristics of TCGA Breast Cancer dataset.**

| **Variable** | **Category** | **All (N=256)** |
| --- | --- | --- |
| **Age at diagnosis (years)** | Median (IQR) | 60.0 (50.5-68.0) |
| **Tumor - N(%)** | Missing values | 3 |
|  | T1 | 51 (20.2%) |
|  | T2 | 154 (60.9%) |
|  | T3 | 34 (13.4%) |
|  | T4 | 14 (5.5%) |
| **Lymph Nodes - N(%)** | Negative | 126 (49.2%) |
|  | Positive | 130 (51.8%) |
| **Metastasis - N(%)** | Missing values | 4 |
|  | Negative | 243 (96.4%) |
|  | Positive | 9 (3.6%) |
| **Estrogen Receptor - N(%)** | Missing values | 3 |
|  | Negative | 57 (22.5%) |
|  | Positive | 196 (77.5%) |
| **Progesterone Receptor - N(%)** | Missing values | 4 |
|  | Negative | 88 (34.9%) |
|  | Positive | 164 (65.1%) |
| **HER2 status - N(%)** | Missing values | 4 |
|  | Negative | 217 (86.1%) |
|  | Positive | 35 (13.9%) |
| **PAM50 Classification – N(%)** | Missing Values | 5 |
|  | Luminal A | 106 (42.2%) |
|  | Luminal B | 73 (29.1%) |
|  | Basal Like | 43 (17.1%) |
|  | HER2 | 29 (11.6%) |
| **Overall Survival (months)** | Median (IQR) | 19.1 (9.0-44.5) |
|  | Range | 0.02-234.09 |

IQR: Interquartile range (i.e. first-third quartiles)

**Supplemental Table 4. Methylation status distribution at the three *MIR9* promoter regions in tumours as compared with normal breast tissues.**

| **Promoters** | | **Tumors**  **(N=101)** | **NBTs**  **(N=10)** | **p-value*** |
| --- | --- | --- | --- | --- |
| *MIR9-1* | M | **43 (42.6%)** | **0 (0%)** | **0.006** |
|  | UM | **58 (57.4%)** | **10 (100%)** |  |
| *MIR9-2* | M | 58 (57.4%) | 5 (50.0%) | 0.744 |
|  | UM | 43 (42.6%) | 5 (50.0%) |  |
| *MIR9-3* | M | 71 (70.3%) | 4 (40.0%) | 0.075 |
|  | UM | 30 (29.7%) | 6 (60.0%) |  |

Analyses performed in 101/131 patients with tumor and in 10/12 NBTs.

*Fisher exact test; M=methylation; UM=unmethylation.

**Supplemental Table 5.** Methylation status distribution at the three combined promoters in tumours as compared with normal breast tissues.

| ***MIR9-1*** | ***MIR9-2*** | ***MIR9-3*** | **Tumors**  **(N=101)** | **NBTs**  **(N=10)** | **p-value*** |
| --- | --- | --- | --- | --- | --- |
| M | M | M | 25 (24.8%) | 0 (0) | 0.098 |
| M | M | UM | 5 (4.9%) | 0 (0) |
| M | UM | M | 10 (9.9%) | 0 (0) |
| M | UM | UM | 3 (3.0%) | 0 (0) |
| UM | M | M | 17 (16.8%) | 3 (30.0%) |
| UM | M | UM | 11 (10.9%) | 2 (20.0%) |
| UM | UM | M | 19 (18.8%) | 1 (10.0%) |
| UM | UM | UM | 11 (10.9%) | 4 (40.0%) |

Analyses performed in 101/131 patients with tumor and in 10/12 NBTs.

*Fisher exact test; M=methylation; UM=unmethylation.

**Supplemental Table 6. Difference of pri-miRNA expression levels in tumour and Normal breast tissues samples.**

|  | **N** | **Tumor sample** | **N** | **NBT** | **p-value** |
| --- | --- | --- | --- | --- | --- |
| pri-miR-9-1 | 113 | 4.25 (1.39-12.57) | 8 | 25.11 (9.53-82.21) | 0.003^*^ |
| pri-miR-9-2 | 113 | 1.36 (0.35-3.67) | 9 | 14.57 (3.40-32.34) | 0.001^*^ |
| pri-miR-9-3 | 113 | 2.18 (0.56-8.70) | 9 | 26.79 (14.04-41.35) | 0.003^*^ |

**Supplemental Table 7. Difference of pri-miRNA expression levels between methylated and unmethylated status of *MIR9* promoters in tumours.**

|  | | **N** | **Median (IQR)*** | **p-value** |
| --- | --- | --- | --- | --- |
| pri-miR-9-1 expression vs. *MIR9-1* methylation status | M | 39 | 3.24 (0.88-9.04) | 0.188 |
|  | UM | 51 | 4.25 (1.24-14.09) |  |
| pri-miR-9-2 expression vs. *MIR9-2* methylation status | M | 52 | 0.84 (0.25-3.65) | 0.565 |
|  | UM | 38 | 1.45 (0.55-2.89) |  |
| pri-miR-9-3 expression vs. *MIR9-3* methylation status | M | 64 | 2.11 (0.55-9.59) | 0.335 |
|  | UM | 26 | 1.62 (0.50-5.32) |  |

Analyses performed in 90/131 tumors. Number of available subjects (N); *IQR: Interquartile range (i.e. first-third quartiles); p-values from Student t-test using log-transformed values of pri-miRNA expressions; M=methylation; UM=unmethylation.

**Supplemental Table 8. Difference of pri-miRNA expression levels between methylated and unmethylated status of *MIR9* promoters in normal breast tissues.**

|  | | **N** | **Median (IQR)*** | **p-value** |
| --- | --- | --- | --- | --- |
| pri-miR-9-1 expression vs. *MIR9-1* methylation status | M | 0 | - | - |
|  | UM | 7 | 21.75 (3.57-118.29) |  |
| pri-miR-9-2 expression vs. *MIR9-2* methylation status | M | 5 | 3.40 (1.66-17.14) | 0.085 |
|  | UM | 3 | 37.39 (14.57-118.32) |  |
| pri-miR-9-3 expression vs. *MIR9-3* methylation status | M | 4 | 36.26 (28.98-78.07) | 0.187 |
|  | UM | 4 | 9.67 (2.52-65.89) |  |

Analyses performed in 8/12 normal breast tissues. Number of available subjects (N); *IQR: Interquartile range (i.e. first-third quartiles); p-values from Student t-test using log-transformed values of pri-miRNA expressions; M=methylation; UM=unmethylation.

**Supplemental Table 9.** Efficiency of amplification calculated for each Real-time PCR as follows: E=(10^(-1/slope)-1) using the slope of the standard curve plots of Ct versus log input of cDNA.

|  | **Median efficiency** | **Median slope** |
| --- | --- | --- |
| **miRNA** |  |  |
| **RNU48** | 0,956±0,052 | -3,438±0,129 |
| **hsa-miR9-5p** | 0,976±0,043 | -3,384±0,100 |
| **hsa-miR9-3p** | 0,943±0,032 | -3,470±0,082 |
| **pri-miRNA** |  |  |
| **RPLPO** | 0,941±0,038 | -3,476±0,102 |
| **pri-miR-9-1** | 0,896±0,016 | -3,601±0,049 |
| **pri-miR-9-2** | 0,893±0,017 | -3,609±0,051 |
| **pri-miR-9-3** | 0,914±0,011 | -3,547±0,032 |
| **mRNAs** |  |  |
| **RPLPO** | 0,941±0,028 | -3,472±0,074 |
| **AR** | 0,894±0,026 | -3,606±0,076 |
| **FOXA1** | 0,905±0,020 | -3,572±0,056 |
| **GSK3B** | 0,978±0,026 | -3,375±0,064 |
